# Comparable efficacy and safety of COVID-19 vaccines for patients receiving tegafur–uracil as postoperative adjuvant chemotherapy

**DOI:** 10.1007/s00595-023-02649-1

**Published:** 2023-02-08

**Authors:** Megumi Nishikubo, Yugo Tanaka, Suguru Mitsui, Takefumi Doi, Daisuke Hokka, Wataru Hojo, Hironori Sakai, Yohei Funakoshi, Kimikazu Yakushijin, Goh Ohji, Hironobu Minami, Yoshimasa Maniwa

**Affiliations:** 1 Division of Thoracic Surgery, Department of Surgery, Kobe University Hospital and Graduate School of Medicine, 7-5-2 Kusunoki-cho, Chuou-ku, Kobe, Hyogo 650-0017 Japan; 2 R&D, Cellspect Co., Ltd., 2-4-23 Kitaiioka, Morioka, Iwate 020-0857 Japan; 3 Division of Medical Oncology/Hematology, Department of Medicine, Kobe University Hospital and Graduate School of Medicine, 7-5-2 Kusunoki-cho, Chuou-ku, Kobe, Hyogo 650-0017 Japan; 4 Division of Infection Disease Therapeutics, Department of Microbiology and Infectious Diseases, Kobe University Hospital and Graduate School of Medicine, 7-5-2 Kusunoki-cho, Chuou-ku, Kobe, Hyogo 650-0017 Japan

**Keywords:** Adjuvant chemotherapy, COVID-19, Non-small-cell lung carcinoma, UFT, Vaccines

## Abstract

**Purpose:**

Many effective vaccines against severe acute respiratory syndrome coronavirus 2 (SARS-CoV-2) have been developed, but a weaker response in individuals undergoing anticancer treatment has been reported. This study evaluates the immunogenic status and safety of SARS-CoV-2 vaccines for patients with non-small-cell lung cancer (NSCLC), receiving tegafur–uracil (UFT) as postoperative adjuvant chemotherapy.

**Methods:**

The subjects of this prospective study were 40 patients who underwent surgery for NSCLC and received SARS-CoV-2 vaccines postoperatively. We compared the antibody titers of SARS-CoV-2 vaccines and the adverse events between patients who received adjuvant UFT and patients who did not.

**Results:**

The mean anti-S1 IgG titers were not significantly different between the UFT and without-UFT groups (mean optimal density, 0.194 vs. 0.205; *P* = 0.76). Multivariate analysis identified the period after the second vaccination as an independent predictor of anti-S1 IgG titer (*P* = 0.049), but not the UFT status (with or without-UFT treatment; *P* = 0.47). The prevalence of adverse events did not differ significantly between the groups, and no severe adverse events occurred.

**Conclusions:**

The efficacy and safety of the SARS-CoV-2 vaccines for NSCLC patients who received postoperative adjuvant UFT chemotherapy were comparable to those for NSCLC patients who did not receive postoperative adjuvant UFT chemotherapy.

**Clinical trial registration:**

This study was registered with the University Hospital Medical Information Network (UMIN) in Japan (UMIN000047380).

## Introduction

The coronavirus disease 2019 (COVID-19), caused by the severe acute respiratory syndrome coronavirus 2 (SARS-CoV-2), is a highly transmissible disease that has resulted in a global pandemic [1]. Severe illness can occur in healthy individuals of any age, but is more common in patients affected by comorbidities, such as chronic disease or cancer [2]. Patients with lung cancer have a higher risk of infection and severe complications than the general population [3], especially among those with a smoking history or comorbidity, such as chronic obstructive pulmonary disease (COPD) [4].

Ten vaccines have received Emergency Use Listing (EUL) status by the World Health Organization (WHO) [5]. In Japan, the approved vaccines are SARS-CoV-2 BNT162b2, mRNA-1273, and ChAdOx1-nCov2, the efficacy and safety of which have been established in phase 3 studies [6–8]. However, several studies have found that individuals taking immunosuppressive agents or anticancer treatments had a lower response to SARS-CoV-2 vaccines [9, 10].

Tegafur–uracil (UFT) therapy has been reported to improve the survival of patients with completely resected pathological stage IA–IIA non-small-cell lung cancer (NSCLC) [11]. These patients take UFT orally twice daily for 2 years. This regimen is used widely in some countries, including Japan; however, the relationship between UFT and the efficacy of COVID-19 vaccination has not been examined. This prospective study evaluated the serological status and safety of the COVID-19 vaccines in patients with NSCLC who were receiving UFT as postoperative adjuvant chemotherapy, compared with patients who did not receive postoperative adjuvant chemotherapy.

## Methods

### Study design

This was a single-center prospective observational study conducted at Kobe University Hospital. The protocol was implemented in accordance with the principles of the Declaration of Helsinki, with approval from the Clinical Research Area Ethics Committee of Kobe University Graduate School of Medicine (B2156703, approved on July 16, 2021). All patients provided written informed consent before inclusion in this study.

### Study population

Patients aged over 20 years old who had undergone complete surgical resection of NSCLC at our institution were eligible for inclusion in this study. Patients receiving UFT as postoperative adjuvant chemotherapy and control patients who underwent surgery for NSCLC and did not receive UFT were enrolled. Written informed consent was obtained from all patients. The exclusion criteria were a history of COVID-19, current treatment for another infectious disease, immunosuppressive agents, or treatment for other malignant diseases. Patients answered questionnaires to assess if there were any adverse events after receiving the first and second dose of the COVID-19 vaccines. Between June and October 2021, 20 NSCLC patients who received UFT as postoperative chemotherapy (UFT group) and 20 NSCLC patients who did not receive UFT (without-UFT group) postoperatively were enrolled in the study.

Among the patients who received UFT, 250 mg of tegafur per square meter of body-surface area per day was administered orally for 2 years. In patients who exhibited adverse reactions, the dose of UFT was reduced.

### Sample collection and measurement of antibody titers

Patients were vaccinated with two doses of the COVID-19 vaccines as per the package insert. Peripheral blood samples were collected 2 weeks to 3 months after the second dose. Serum samples were obtained by centrifuging the blood samples for 5 min at 2500 rpm at room temperature and then stored at −80 °C.

We measured the IgG against the S1 subunit of spike protein (S1 protein) to evaluate the efficacy of the vaccine against the nucleocapsid protein (N protein) to investigate for prior infection with SARS-CoV-2. To measure the antibody titers against the S1 and N proteins, we used the QuaResearch COVID‐19 Human IgM IgG ELISA Kit (Spike Protein‐S1) and QuaResearch COVID‐19 Human IgM IgG ELISA Kit (Nucleocapsid Protein) (RCOEL961S1 and RCOEL961N, respectively; Cellspect Co. Ltd.). These kits are based on the indirect ELISA method and include immobilized recombinant antigenic proteins (S1 and N proteins: S1, 251–660 AA; N, 1–419 AA) of SARS‐CoV‐2 expressed in *Escherichia coli*. Serum samples were diluted at 1:1000 in 1% bovine serum albumin/phosphate-buffered saline containing Tween‐20 (PBS-T). The plates were read at 450 nm on an automated ELISA system (QRC5LB925; Cellspect Co. Ltd.) according to the manufacturer’s measurement protocol. The OD cut‐off value was set at 0.7 for anti-N IgG, based on the study of the differences in antibody responses between patients with COVID-19 and negative-control individuals (without COVID-19).

### Adverse events

Local or systemic adverse events and the use of antipyretic analgesics were investigated using a self-administered questionnaire. Fever was defined as a temperature of 38 °C or higher [ ≥ Grade 1 in Common Terminology Criteria for Adverse Events (CTCAE) v5.0].

### Statistical analysis

The primary endpoint was the titer of the COVID-19 vaccine-induced IgG and the secondary endpoints were the existence of local or systemic adverse events and their severity. Welch’s *t* test was used to compare continuous variables, whereas Fisher’s exact test was applied to compare nominal variables. A multiple regression analysis was used for multivariate analysis. Age and the period after the second vaccination were concluded to contribute to the waning IgG antibody level, in accordance with previous studies [12, 13]. Therefore, these factors were chosen as explanatory variables in the multiple regression analysis. All statistical analyses were performed using R (The R Foundation for Statistical Computing, Vienna, Austria). Significance was set at *P* < 0.05.

## Results

### Patient characteristics

Table 1 summarizes the clinical characteristics of the patients. The UFT group comprised 8 (40%) men and 12 (60%) women (mean age, 67.7 ± 9.2 years); the without-UFT group comprised 10 (50%) women and 10 (50%) men (mean age, 67.4 ± 8.5 years). The pathological stages differed between the two groups. The mean period after the second vaccination dose was 50.1 ± 21.2 days in the UFT group and 58.4 ± 21.3 days in the without-UFT group. UFT therapy was suspended for several days around the vaccination date in 8 of the 20 UFT group patients.

**Table 1:** Clinicopathological characteristics of the two groups of patients

Characteristics	Without-UFT group (*n* = 20)	UFT group (*n* = 20)	SMD
Mean age, y (SD)	67.4 (8.5)	67.7 (9.2)	0.03
Sex			
Male	10 (50)	8 (40)	0.20
Female	10 (50)	12 (60)	
Type of vaccination			
BNT162b2 (Pfizer)	19 (95)	19 (95)	0
mRNA-1273 (Moderna)	1 (5)	1 (5)	
Pathological stage (UICC 8th)			
0	1 (5)	0 (0)	3.10
IA1	4 (20)	0 (0)	
IA2	13 (65)	3 (15)	
IA3	0 (0)	9 (45)	
IB	0 (0)	7 (35)	
IIA	1 (5)	1 (5)	
IIB	1 (5)	0 (0)	
Histology			
Adenocarcinoma	18 (90)	17 (85)	0.50
Squamous carcinoma	1 (5)	2 (10)	
Adenosquamous carcinoma	0 (0)	1 (5)	
Pleomorphic carcinoma	1 (5)	0 (0)	
Type of surgery			
Bilobectomy	0 (0)	1 (5)	0.46
Lobectomy	19 (95)	19 (95)	
Segmentectomy	1 (5)	0 (0)	
Smoking history			
Ex-smokers	12 (60)	12 (60)	0
Never smokers	8 (40)	8 (40)	
Mean period after the vaccination, d (SD)	58.4 (21.3)	50.1 (21.2)	0.39
Mean period after surgery, m (SD)	14.4 (14.3)	10.4 (6.0)	0.36
Mean period of receiving UFT, m (SD)	NA	9.1 (6.2)	

Values are expressed as *n* (%) unless otherwise indicated

*NA* not available, *SD* standard deviation, *SMD* standardized mean difference, *UFT* tegafur–uracil

### Serological outcomes

**Fig. 1:**
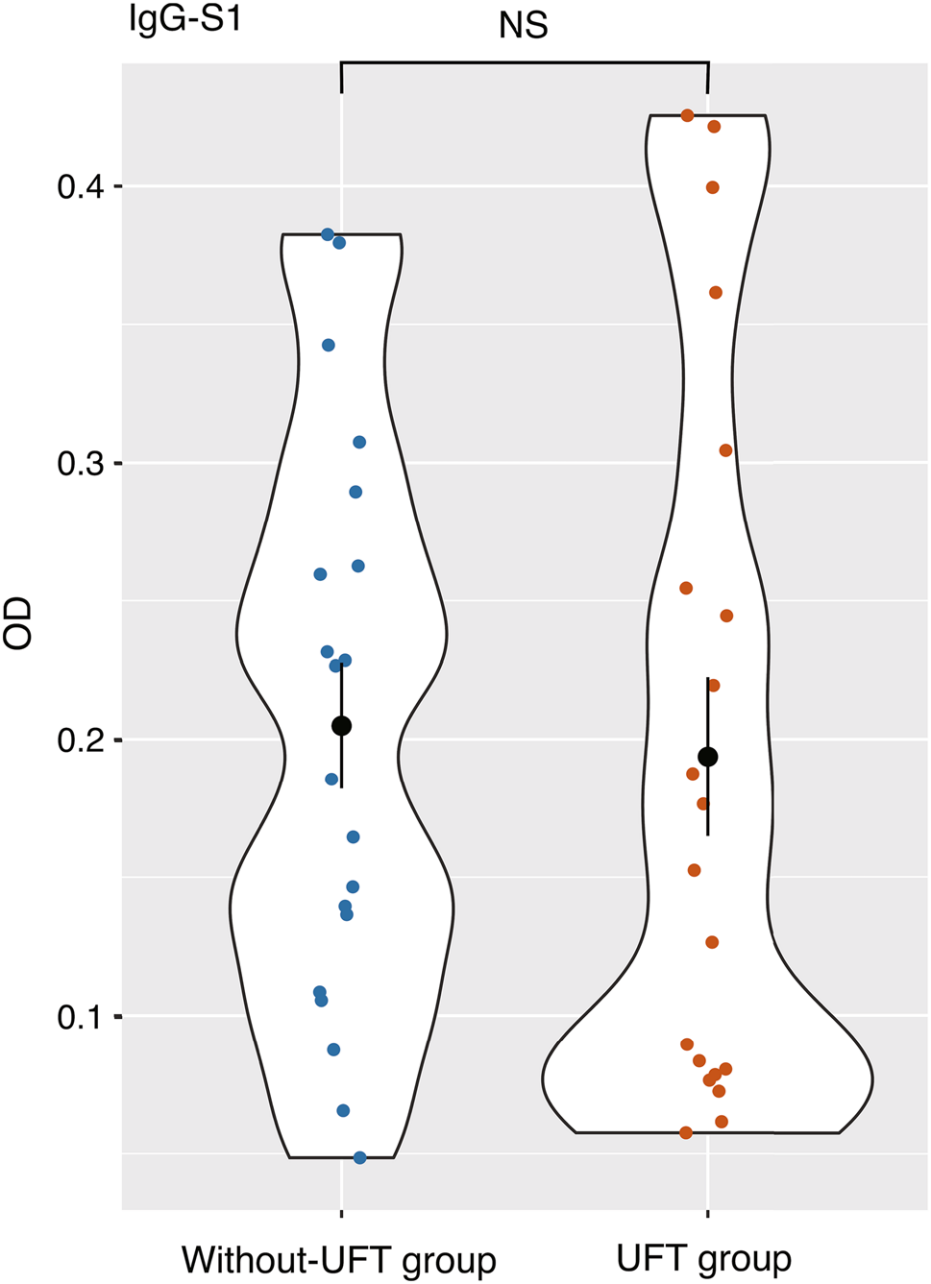
Violin plot of the anti-S1 IgG titers. The mean titers of anti-S1 IgG after the second dose were not significantly different between the tegafur–uracil (UFT) and without-UFT groups (mean optimal density 0.194 ± 0.129 vs. 0.205 ± 0.102; *P* = 0.76). *OD* optical density, *UFT* tegafur–uracil, *NS* not significant

**Fig. 2:**
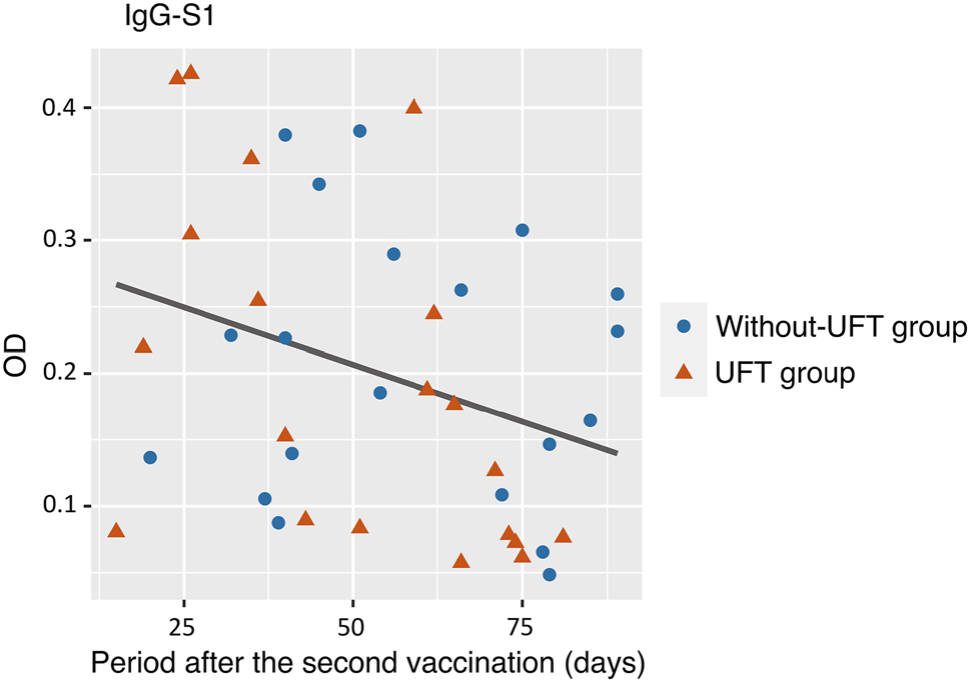
Scatter plot and regression lines of the anti-S1 IgG titer and the period after the second vaccination. There was a negative correlation between these parameters. *OD* optical density, *UFT* tegafur–uracil

All patients had anti-N IgG levels below the cutoff value, indicating that none were infected with SARS-CoV-2 before the sample collection. The mean titers of anti-S1 IgG after the second dose were not significantly different between the UFT and without-UFT groups (mean optimal density, 0.194 ± 0.129 vs. 0.205 ± 0.102; *P* = 0.76; Fig. 1). The period after the second vaccination and the anti-S1 IgG level were negatively correlated, as shown in Fig. 2. The multivariate analysis of age, UFT status (with or without-UFT therapy), and the period after the second vaccination revealed that the period after the second vaccination was an independent predictor of the anti-S1 IgG titer (*P* = 0.049), whereas the UFT status was not (*P* = 0.47) (Table 2).

**Table 2:** Multiple regression analysis for the prediction of S1 IgG titers

Factors	Estimate	Standard error	*t* value	*P* value
Age	0.0011	0.0021	0.547	0.59
UFT	− 0.026	0.036	− 0.723	0.47
Period after the second vaccination	− 0.0017	0.00087	− 2.038	0.049

*UFT* tegafur–uracil

Regarding the temporary discontinuation of UFT, the mean titers of anti-S1 IgG after the second dose were not significantly different between the 8 patients whose UFT was suspended and the 12 patients who continued receiving UFT without interruption (mean optimal density, 0.181 ± 0.137 vs. 0.202 ± 0.128; *P* = 0.74).

### Adverse events

Table 3 summarizes the adverse events that occurred after vaccination. Fourteen (70%) of the 20 UFT group patients and 17 (85%) of the without-UFT group patients suffered at least one adverse event (*P* = 0.45). However, no severe adverse event was recorded in either group. According to the medical records, no patient had suffered COVID-19 as of the end of June 2022 (7 months after enrollment of the last patient).

**Table 3:** Prevalence of adverse events after receiving COVID-19 vaccinations

Characteristics	Without-UFT group (*n* = 20)	UFT group (*n* = 20)	*P* value
At least one adverse event	17 (85)	14 (70)	0.45
Dose 1			
Local events			
Local pain	11 (55)	9 (45)	
Local swelling	6 (30)	3 (15)	
Systemic events			
Fever	0 (0)	0 (0)	
Fatigue	4 (20)	2 (10)	
Headache	2 (10)	1 (5)	
Chills	0 (0)	1 (5)	
Nausea	0 (0)	0 (0)	
Vomiting	0 (0)	0 (0)	
Diarrhea	0 (0)	1 (5)	
Myalgia	0 (0)	0 (0)	
Arthralgia	1 (5)	1 (5)	
Anaphylaxis	0 (0)	0 (0)	
Anti-inflammatory agent	1 (5)	1 (5)	
Dose 2			
Local events			
Local pain	12 (60)	11 (55)	
Local swelling	5 (25)	4 (20)	
Systemic events			
Fever	1 (5)	3 (15)	
Fatigue	4 (20)	7 (35)	
Headache	3 (15)	1 (5)	
Chills	0 (0)	2 (10)	
Nausea	0 (0)	0 (0)	
Vomiting	0 (0)	0 (0)	
Diarrhea	0 (0)	0 (0)	
Myalgia	0 (0)	0 (0)	
Arthralgia	1 (5)	0 (0)	
Anaphylaxis	0 (0)	0 (0)	
Anti-inflammatory agent	2 (10)	5 (25)	

Values are expressed as *n* (%) of patients

## Discussion

In this study cohort of NSCLC patients, UFT did not affect the anti-S1 antibody titers after COVID-19 vaccination, temporary discontinuation of UFT therapy did not affect the anti-S1 antibody titers after COVID-19 vaccination, and no severe adverse events ( **≥ **Grade 3 in CTCAE v5.0) occurred.

First, UFT did not affect the immunogenicity of the COVID-19 vaccines. Previous studies have shown that the antibody titers are correlated with the effectiveness of protection at a population level, but the threshold of the antibody titers that can protect individuals from infection is unknown [14, 15]. A prospective study aimed at examining healthy individuals who received COVID-19 vaccines to characterize their antibody titers revealed that the antibody levels decreased with time [16]. This is consistent with the results shown in Fig. 2. A multivariate analysis performed to eliminate this effect revealed that receiving UFT was not an independent predictive factor of anti-S1 IgG titer.

Second, temporary discontinuation of UFT did not affect the efficacy of the vaccines. UFT prevents the postoperative recurrence of NSCLC and is a time-dependent agent. Therefore, maintaining the blood level of UFT is important to achieve the optimal effect [11]. This study demonstrated that patients can receive COVID-19 vaccines without discontinuation of UFT therapy.

Finally, no severe adverse events occurred in this cohort. The prevalence of local swelling was higher in this study than in the cohort aged > 55 years of the phase 3 BNT162b2 mRNA COVID-19 vaccine study. However, other adverse events were equivalent or less frequent/severe. This might be explained by the older age of our cohort. There was no significant difference in the incidence of adverse events between the UFT group and the without-UFT group; hence, the COVID-19 vaccines seem to be safe, even for patients receiving UFT therapy. These results led us to propose that patients with NSCLC, who are being treated with UFT therapy postoperatively, can receive COVID-19 vaccination like the healthy population, and that temporary discontinuation of the UFT regimen because of COVID-19 vaccination is unnecessary. To our knowledge, this is the first report on the relationship between UFT therapy and the efficacy of COVID-19 vaccines.

The antibody titers of our without-UFT group were slightly lower than those of healthy volunteers enrolled in a previous study that used the same antibody-measuring kit. In our data, peripheral blood samples were collected 2 weeks to 3 months after the second vaccination. Conversely, in the previous study, the blood samples were collected 2 weeks after the second vaccination [17], which may explain the discrepancies. Alternatively, the postoperative status might affect immunogenicity.

UFT therapy is composed of tegafur and uracil. Tegafur is a prodrug of fluorouracil (5-FU), which inhibits the synthesis of thymidine. Methotrexate (MTX) has a similar mechanism of action as an inhibitor of pyrimidine synthesis. The relationship between MTX and the efficacy of COVID-19 vaccines has been described extensively. Several previous studies found that MTX affects the immune response, whereas others found that patients receiving MTX showed similar neutralizing antibody titers to healthy controls [18, 19]. The relationship between other fluoropyrimidines, such as TS-1, including tegafur and gemcitabine (a fluorinated drug of cytosine), and the efficacy of COVID-19 vaccines has not been reported. In Japan, TS-1 is given frequently for gastric, colon, and breast cancer, and these findings would be useful in these fields. Further research is needed to establish whether the present results can be extrapolated to these drugs.

This study had several limitations. First, the number of patients was small. Although there were no significant differences in the mean titer of anti-S1 IgG between the UFT and the without-UFT groups, further studies on a larger cohort are required to confirm whether UFT affects the efficacy of vaccination. Second, we investigated the titers of antibodies. Several studies have reported a correlation between the titer of antibodies and the effect of infection prevention; however, the threshold is unknown [14, 15]. Thus, it is not guaranteed that antibody titers can be used as a surrogate marker of vaccine efficacy. In this series, none of the patients had suffered COVID-19; however, the effects of third and fourth vaccinations would be too complex to compare. Finally, we evaluated only the humoral response of the COVID-19 vaccine. Although current vaccination efforts have focused on the induction of neutralizing antibodies to SARS-CoV-2, T-cell immunity may be essential to protect the patient from infection. The phase 1 and phase 2 studies of the vaccines analyzed the T-cell response and found high cytokine secretion in humans [20]. However, the extent to which this response contributes to protecting the patient from infection clinically remains unclear. Several studies have reported the effect of T-cell response in patients who received chemotherapy with anti-CD20 antibody or immunosuppression, considering that the seroconversion rate was much lower than that of the general population [21, 22]. Thus, the relationship between UFT and T-cell response warrants further investigation.
